# Huangqin Decoction Exerts Beneficial Effects on Rotenone-Induced Rat Model of Parkinson's Disease by Improving Mitochondrial Dysfunction and Alleviating Metabolic Abnormality of Mitochondria

**DOI:** 10.3389/fnagi.2022.911924

**Published:** 2022-07-15

**Authors:** Li Gao, Min Cao, Guan-hua Du, Xue-mei Qin

**Affiliations:** ^1^Modern Research Center for Traditional Chinese Medicine, The Key Laboratory of Chemical Biology and Molecular Engineering of Ministry of Education, Shanxi University, Taiyuan, China; ^2^Key Laboratory of Effective Substances Research and Utilization in TCM of Shanxi Province, Taiyuan, China; ^3^Peking Union Medical College, Institute of Materia Medica, Chinese Academy of Medical Sciences, Beijing, China

**Keywords:** Huangqin Decoction, Parkinson's disease, mitochondria, ketone bodies, aerobic glycolysis

## Abstract

Parkinson's disease (PD) is a common neurodegenerative disease, and the pathogenesis of PD is closely related to mitochondrial dysfunction. Previous studies have indicated that traditional Chinese medicine composition of Huangqin Decoction (HQD), including *Scutellariae Radix, licorice*, and *Paeoniae Radix Alba*, has therapeutic effects on PD, but whether HQD has a therapeutic effect on PD has not been reported. In this study, the protective effects of HQD on rotenone-induced PD rats were evaluated by behavioral assays (open field, rotating rod, suspension, gait, inclined plate, and grid) and immunohistochemistry. The mechanisms of HQD on attenuation of mitochondrial dysfunction were detected by biochemical assays and mitochondrial metabolomics. The results showed that HQD (20 g/kg) can protect rats with PD by improving motor coordination and muscle strength, increasing the number of tyrosine hydroxylase (TH)-positive neurons in rats with PD. Besides, HQD can improve mitochondrial dysfunction by increasing the content of adenosine triphosphate (ATP) and mitochondrial complex I. Mitochondrial metabolomics analysis revealed that the ketone body of acetoacetic acid (AcAc) in the rotenone group was significantly higher than that of the control group. Ketone bodies have been known to be used as an alternative energy source to provide energy to the brain when glucose was deficient. Further studies demonstrated that HQD could increase the expression of glucose transporter GLUT1, the content of tricarboxylic acid cycle rate-limiting enzyme citrate synthase (CS), and the level of hexokinase (HK) in rats with PD but could decrease the content of ketone bodies [AcAc and β-hydroxybutyric acid (β-HB)] and the expression of their transporters (MCT1). Our study revealed that the decrease of glucose metabolism in the rotenone group was parallel to the increase of substitute substrates (ketone bodies) and related transporters, and HQD could improve PD symptoms by activating the aerobic glycolysis pathway.

## Introduction

Parkinson's disease (PD) is a common neurodegenerative disease in middle-aged and elderly people (Goel et al., 2021), which is characterized by many motor symptoms (Dorsey et al., 2018; Zheng et al., 2019b), such as resting tremor, muscle rigidity, bradykinesia, abnormal posture, and gait, as well as non-motor symptoms, including mood and mental changes, autonomic dysfunction, sleep disorders, sensory disturbances, and cognitive decline (Jankovic, 2008; Hu et al., 2017; Zhang et al., 2018b). Dopamine (DA) deficiency caused by degeneration and necrosis of dopaminergic neurons in substantia nigra (SN) is the main cause of PD (Kang et al., 2019).

The DA replacement therapy is still the most effective method for the treatment of dyskinesia, but it cannot prevent the progression of PD, and sustained efficacy requires an increase in dose, which often leads to side effects (Hu et al., 2017). Traditional Chinese medicine adopts a dialectical method to treat PD, which can improve the patient's quality of life to a certain extent. Huangqin Decoction (HQD) is derived from “Treatise on Febrile Diseases” (Gao et al., 2019; Li et al., 2020). The whole prescription includes 9 g of *Scutellariae Radix*, 3 g of *licorice*, 9 g of *Paeoniae Radix Alba*, and 20 g of *Jujubae Fructus*. HQD is usually used to treat digestive system diseases (Wang et al., 2017) and has anti-inflammatory, antioxidant, and anti-ulcer effects (Zhang et al., 2008; Zou et al., 2015).

Previous pharmacological studies have shown that the composed herbs of HQD have a good therapeutic effect on PD (Zhang et al., 2017a). Total flavonoids of *Scutellaria baicalensis* Georgi and total glucosides of paeony extracted from Radix Paeoniae Alba can improve the behavior of N-methyl-4-phenyl-1,2,3,6-tetrahydropyridine (MPTP)-induced PD mice and can increase the number of dopaminergic neurons in SN (Li et al., 2016; Zheng et al., 2019a). Another study has shown that baicalein, an active component isolated from *S. baicalensis* Georgi, can effectively improve the behaviors of MPTP-induced PD mice, rotenone-induced PD rats, and 6-hydroxydopamine-induced PD rats (Gao et al., 2015a; Zhang et al., 2017b; Zhu et al., 2019). Furthermore, licorice intake can improve the symptoms of patients with PD (Petramfar et al., 2020). However, whether HQD has protective effects on PD is still unclear, which is worthy of further study.

The exact pathogenesis of PD is unclear, but it has been convincingly recognized for many years that mitochondrial dysfunction plays a crucial role in the occurrence and development of dopaminergic degeneration (Ludtmann and Abramov, 2018). Mitochondria are organelles that produce adenosine triphosphate (ATP), which is the main source of cell energy. The metabolism of the brain is highly dynamic, and the demand for energy is extremely high. The impaired mitochondrial function will lead to decreased ATP production, impaired bioenergy, apoptosis, and oxidative stress (Hroudová and Fišar, 2011). Blockage of mitochondrial pathways will lead to the accumulation of a large number of damaged mitochondria, resulting in an insufficient supply of cellular energy, eventually leading to the gradual death of neurons and the progression of PD. Furthermore, the brain of patients with PD showed mitochondrial complex I deficiency, and the pathogenic genes causing recessive PD are involved in mitochondrial function and quality control, which further proved that mitochondrial dysfunction plays an important role in the pathogenesis of PD (Holper et al., 2019).

In many cases, energy deficiency and decreased ATP levels were observed in PD (Saxena, 2012). Aging is the main risk factor of PD, which damages brain glucose metabolism and reduces mitochondrial biosynthesis and ATP level (Hoyer, 1990). In addition, glycolysis and mitochondrial function are decreased in patients with PD (Schapira, 1993; Hsu et al., 2017). Under normal physiological conditions, the brain mainly uses glucose to generate ATP (Leino et al., 2001), and glucose is converted into pyruvic acid by aerobic glycolysis and, finally, into ATP for the brain to use. However, in the absence of glucose, ketone bodies become an important energy source for the brain (Norwitz et al., 2019; Jensen et al., 2020). Ketone bodies are closely related to mitochondrial bioenergy and mitochondrial dynamics (Veech, 2004; Thai et al., 2021), which can improve the pathological damage and mitochondrial function of dopaminergic neurons in SN of animals with PD, increase the production of ATP, and alleviate the energy crisis caused by the decrease of brain glucose metabolism in neurodegenerative diseases such as PD.

Rotenone can block the function of mitochondrial complex I, enhance oxidative stress (Henchcliffe and Beal, 2008; Stykel et al., 2018), and induce the accumulation of α-synuclein (Cen et al., 2021), resulting in mitochondrial dysfunction and irreversible PD-like symptoms. In this study, the dopaminergic neurodegeneration in rats with PD was induced by long-term systemic injection of rotenone, and its pathological characteristics were observed. The potential therapeutic effect of HQD was evaluated in rotenone-induced PD rats, and the underlying mechanisms were investigated by the detection of mitochondrial function and metabolic alterations in mitochondrial.

## Materials and Methods

### Ethics

All experiments were performed under the NIH Guidelines for Care and Use of Laboratory Animals (USA) and were approved by the Animal Ethics Committee of Shanxi University.

### Materials

The BCA kit, DA (rat) kit, β-hydroxybutyric acid (β-HB; rat) kit, acetoacetic acid (AcAc; rat) kit, and citrate synthase (CS; rat) kit were purchased from Sangon Biotech (Shanghai, China). The ATP assay kit, mitochondrial complex I assay kit, glucose kit, and hexokinase (HK) kit were purchased from Solarbio (Beijing, China). *Scutellariae Radix, licorice, Paeoniae Radix Alba*, and *Jujubae Fructus* were purchased from the Beijing Tongren drug store (Beijing, China). Rotenone was purchased from Target Mol (USA) and madopar was purchased from Shanghai Roche Pharmaceuticals Ltd. D2O was purchased from Norell (Landisville, PA, USA). Sodium 3-trimethylsilyl 2,2,3,3-d4 propionate (TSP) was purchased from Cambridge Isotope Laboratories Inc. (Andover, MA, USA). Primary antibodies for GLUT1, MCT1, and GAPDH were purchased from Proteintech Group Co., Ltd. (Chicago, IL), and anti-rabbit lgG/HRP and anti-MCT2 were purchased from Bioss, Inc.

### HQD Preparation Method

Huangqin Decoction was prepared by *Scutellariae Radix* (9 g), *Paeoniae Radix Alba* (9 g), *licorice* (3 g), and *Jujubae Fructus* (20 g). The herbs were soaked in water for 30 min and decocted twice in 10 volumes and 5 volumes of distilled water (v/w) for 30 min, respectively, and the decoctions were mixed. The extract was filtered and concentrated to a certain volume to obtain HQD, and the HQD was frozen and stored.

### UPLC-MS/MS Identification of Chemical Components in HQD

Huangqin Decoction powder (0.30 g) was extracted with 20 ml methanol in a water bath at 40°C for 30 min and centrifuged at 4,000 r/min for 5 min. Finally, the supernatant was filtered by a 0.22-μm syringe filter before use.

The main components of HQD were analyzed by ultra-performance liquid chromatography coupled with mass spectrometry (UPLC-MS). The UPLC-MS/MS system consisted of a triple quadrupole mass spectrometer equipped with an electrospray ionization (ESI) source. The mobile phase consisted of 0.1% formic acid (A) water (solvent A) and (B) acetonitrile (solvent B). The gradient elution was as follows: 0–2 min is 5% B, 2–5 min is 5% B to 20% B, 5–15 min is 20% B to 40% B, 15–20 min is 40% B to 90% B, 20–22 min is 90% B, 22–22.5 min is 90% B to 5% B, and 22.5–25 min is 5% B with a flow rate of 0.3 ml/min.

The ESI source was used to detect positive and negative ions simultaneously, with the mass scanning range of m/z 100–1,500 Da, the spray voltage is 3.5 kV in positive ion mode and 2.5 kV in negative ion mode, and the capillary temperature is 320°C.

### Experimental Animals

A total of 72 male Sprague Dawley rats (220–240 g) were purchased from Beijing Vital River Laboratories (Beijing, China). After adapting to the new environment for 1 week, all rats were accommodated at room temperature (20–25°C) and constant humidity (30–60%) and ate and drank freely in light and dark circulation for 12 h.

### Experimental Procedure

**Figure 1:**
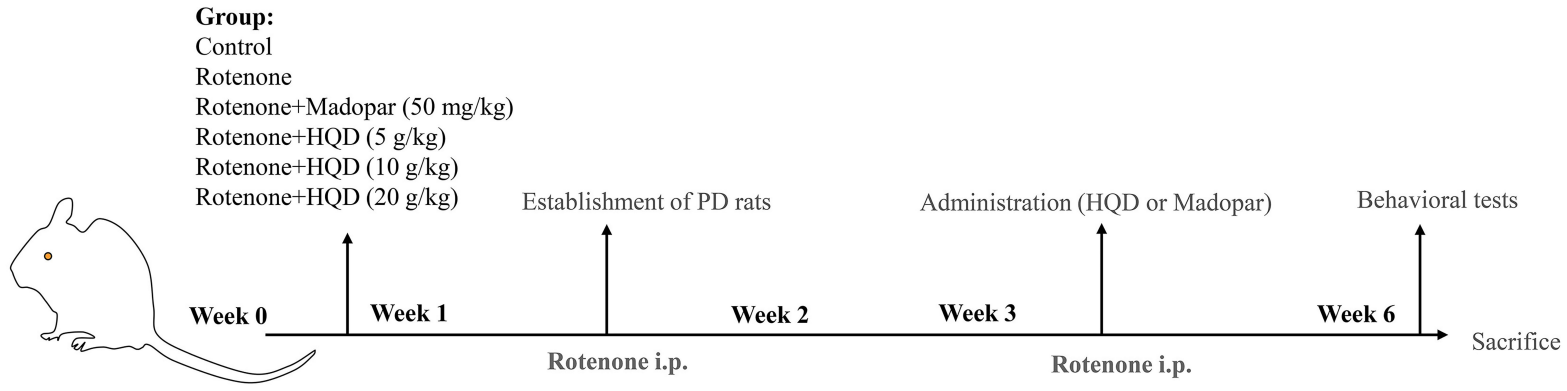
The design of animal experiment.

The 72 rats were divided randomly into 6 groups (*n* = 12 per group), namely, the control group, the model group (rotenone, 2.5 mg/kg), the positive drug group (madopar, 50 mg/kg), the low-dose HQD group (5 g/kg), the medium-dose HQD group (10 g/kg), and the high-dose HQD group (20 g/kg). Rotenone was dissolved in sunflower oil. Rats in the control group were intraperitoneally given sunflower oil, and rats in the other groups were intraperitoneally injected with the same volume of rotenone (2.5 mg/kg) once a day for 6 weeks. From the third week, HQD and madopar were intragastrically administered every day for 4 weeks. The rats in the control group and the rotenone group were intragastrically administered with the same amount of normal saline every day. The rats in each group were subjected to behavioral experiments last week (Figure 1).

### Behavioral Assessment

The effects of HQD on rotenone-induced PD rats were evaluated by open field, rotating rod, suspension, gait, inclined plate, and grid experiments. Open field test, rotating rod experiment, grid experiment, and gait experiment were used to appraise the motor coordination ability of rats with PD. Suspension and inclined plate were used to evaluate the muscle strength of rats with PD.

#### Open Field Test

The animals were subjected to an open field test for 5 min (Peshattiwar et al., 2020). Before each trial, 75% alcohol was used to wipe the bottom of the box to remove the odor and clean up the feces to prevent the residual odor between the two experiments. The rats were placed in the center of the box and allowed to move freely. The statistical parameters include the number of crossing, the escape time from the central grid, and upright times (Yan et al., 2020).

#### Rotating Rod Experiment

The rats were placed on a rotating rod instrument and adapted for 1 min at 5 r/min; then, the instrument was adjusted to 13 r/min for testing (Peshattiwar et al., 2020). The time from the start of rotating to the fall of the rotating rod was measured. The total time is 120 s, and each rat was measured 3 times (Kuribara et al., 1977).

#### Suspension Experiment

The two forepaws of the rats were suspended on a horizontally fixed rope of 40 cm (0.3 cm in diameter and 60 cm in length) from the ground. The time before the rat fell to the ground was recorded and scored, namely, 0–4 s for 0 points, 4–9 s for 1 point, 9–14 s for 2 points, 14–19 s for 3 points, 19–24 s for 4 points, 24–29 s for 5 points, and more than 29 s is recorded as 6 points (Kuribara et al., 1977).

#### Gait Experiment

To obtain footprints, the rats were coated with different nontoxic pigments on their front feet and back feet, and the animals were allowed to walk along a channel with a length of 100 cm and a width of 10 cm (the height of the high wall was 15 cm). A new blank sheet of paper was placed on the floor, and stride length and overlap distance were recorded for analysis. The stride length was obtained by measuring the average distance moving forward between the two strides, and the overlapping distance between the front and back footprints was measured to evaluate the uniformity of step alternation (Rennie et al., 2021). When the center of the back footprint fell above the center of the previous front footprint, it was recorded as zero. When the footprints do not overlap, the distance between the centers of the footprints was recorded for analysis (Zheng et al., 2019b; Haider et al., 2020).

#### Inclined Plate Experiment

The center of the inclined plate device was a rough surface with a width of 15 cm and a length of 33 cm, which forms an angle of 85° with the ground. The rat was put in the center of the rough surface, and the residence time on the inclined plate was recorded for analysis.

#### Grid Experiment

An independent metal grid (grid size 1.2 × 1.2 cm) perpendicular to the horizontal plane was selected as the experimental device. The rat was put in the center of the metal grid, so that the position of the rat is perpendicular to the ground, and the metal grid was grasped with each toe. A stopwatch was used to record the time it takes for the rat to move from a static state to any paw movement, which is the movement latency of the rat.

### Tissue Collection

The rats with PD were sacrificed 12 h after the last injection of rotenone or administration of HQD. After anesthesia, the brain was taken out and the cerebral hemispheres were severed. The striatum, midbrain, and cortex were separated from one hemisphere and rapidly frozen in liquid nitrogen for biochemical research. The other hemispheres were fixed with a 4% paraformaldehyde solution for 2 days until the tissue was completely sunk, and then sectioned.

### Immunohistochemistry of Tyrosine Hydroxylase

The SN was isolated from the midbrain of the rats to prepare paraffin sections. After antigen repair of tissue sections, the slides were placed in phosphate-buffered saline (PBS), and then it was shaken and washed 3 times on a decolorizing shaker. The sections were incubated with a 3% hydrogen peroxide solution, at room temperature in the dark, washed 3 times on a decolorizing shaker, and blocked at room temperature for 30 min. The sections were incubated with antibody tyrosine hydroxylase (TH) overnight at 4°C, rinsed 3 times with phosphate buffer containing Tween 20, incubated with secondary antibody (HRP marker), developed with diaminobenzidine, and examined under a microscope. The proportion of TH-positive neuron area was calculated.

### ELISA Assay of DA Content

After the addition of PBS buffer, the striatum samples were homogenized, the mixture was centrifuged at 3,000 r/min for 20 min, and the supernatant was collected. The corresponding reagents were added according to the instructions of the rat DA ELISA kit. After the reaction, the absorbance of the samples and standards was measured, and the content of DA was calculated.

### Assay of ATP and Mitochondrial Complex I

The cortex samples were homogenized after adding ATP extract, and the mixture was centrifuged at 8,000 g for 10 min. The supernatant was transferred to a centrifuge tube and the chloroform was added. The mixture was centrifuged at 10,000 g for 3 min, and the supernatant was collected. The reagents were added successively according to the instructions. After the reaction, the absorbance of the samples was measured, and the content of the ATP was calculated.

The cortex samples were homogenized after adding mitochondrial complex I extract, and the mixture was centrifuged at 600 g for 10 min. The supernatant was transferred to another centrifuge tube and centrifuged at 11,000 g for 15 min. The extract was added to the precipitation and sonicated. The reagents were added successively according to the instructions. After the reaction, the absorbance of the samples was measured, and the content of the mitochondrial complex I was calculated.

### Mitochondrial Metabolomics Analysis

The midbrain mitochondria were used for metabolomics analysis. The midbrain mitochondria were separated by using the mitochondrial extraction kit. The metabolites were extracted with 1 ml of precooled methanol and then crushed by ultrasound for 5 min. Thereafter, the precipitate was centrifuged and the supernatant was collected, which was dried into powder by a centrifugal concentrator. The powder was dissolved in a 600-μl phosphate buffer and centrifuged, and a 550-μl solution was put into an NMR tube for determination.

### NMR Data Processing

Data were obtained on a Bruker 600-MHz Avance III NMR spectrometer, and the spectra were recorded by NOESY pulse sequence. The MestReNova software (Spain) was used to process the spectrum, the spectral phase was adjusted, and baseline correction was performed. The peak position was calibrated with TSP as zero, the water peak was removed, and the range of δ 0.00–7.40 ppm was divided at intervals of δ 0.04 ppm. The collected spectra were integrated, and the integrated data were collated for multivariate statistical analysis.

### Content Determination of Representative Metabolites (β-HB, AcAc, and Glucose) and Enzymes (CS and HK)

The ELISA kit was used to determine the contents of β-HB, AcAc, and CS in mitochondria of rat midbrain. The mitochondria precipitation was crushed by ultrasonic after adding PBS, the mixture was centrifuged at 3,000 r/min for 20 min, and the supernatant was collected. The corresponding reagents were added according to the instructions of the rat ELISA kit. After the reaction, the absorbance of the samples and standards was measured, and the contents of β-HB, AcAc, and CS were calculated.

The content of glucose and HK in the striatum was measured by kit. Distilled water and HK extract were added into the striatum to determine glucose and HK levels, respectively. The mixture was homogenized, and the striatum sample used to measure the glucose content was boiled in a boiling water bath for 10 min and centrifuged at 8,000 g for 10 min. The striatum samples used to measure HK level were directly centrifuged at 8,000 g for 10 min. The supernatant of the two samples was collected. The corresponding reagents were added according to the instructions of the glucose and HK kit. After the reaction, the absorbance of the samples was measured, and the content of glucose and HK was calculated.

### Western Blot

The midbrain mitochondria and striatum of the rat brain were collected and dissolved in RIPA lysis buffer containing 1% PMSF, and the total protein solution was collected by centrifugation. The protein solution was separated by sodium dodecyl sulfate (SDS) polyacrylamide gel electrophoresis and the protein was transferred to a polyvinylidene fluoride membrane. The membrane was incubated with antibodies GLUT1 (1:1,000), MCT1 (1:3,000), MCT2 (1:1,000), and GAPDH (1:1,000) overnight and also with lgG/HRP rabbit antibody (1:5,000), and the protein signal was collected by chemiluminescence.

### Statistical Analysis

All data are represented as the mean ± SEM. The data were statistically analyzed by one-way ANOVA or Student's *t* test. The value of *P* < 0.05 was regarded as statistically significant.

## Results

### Chemical Analysis

**Figure 2:**
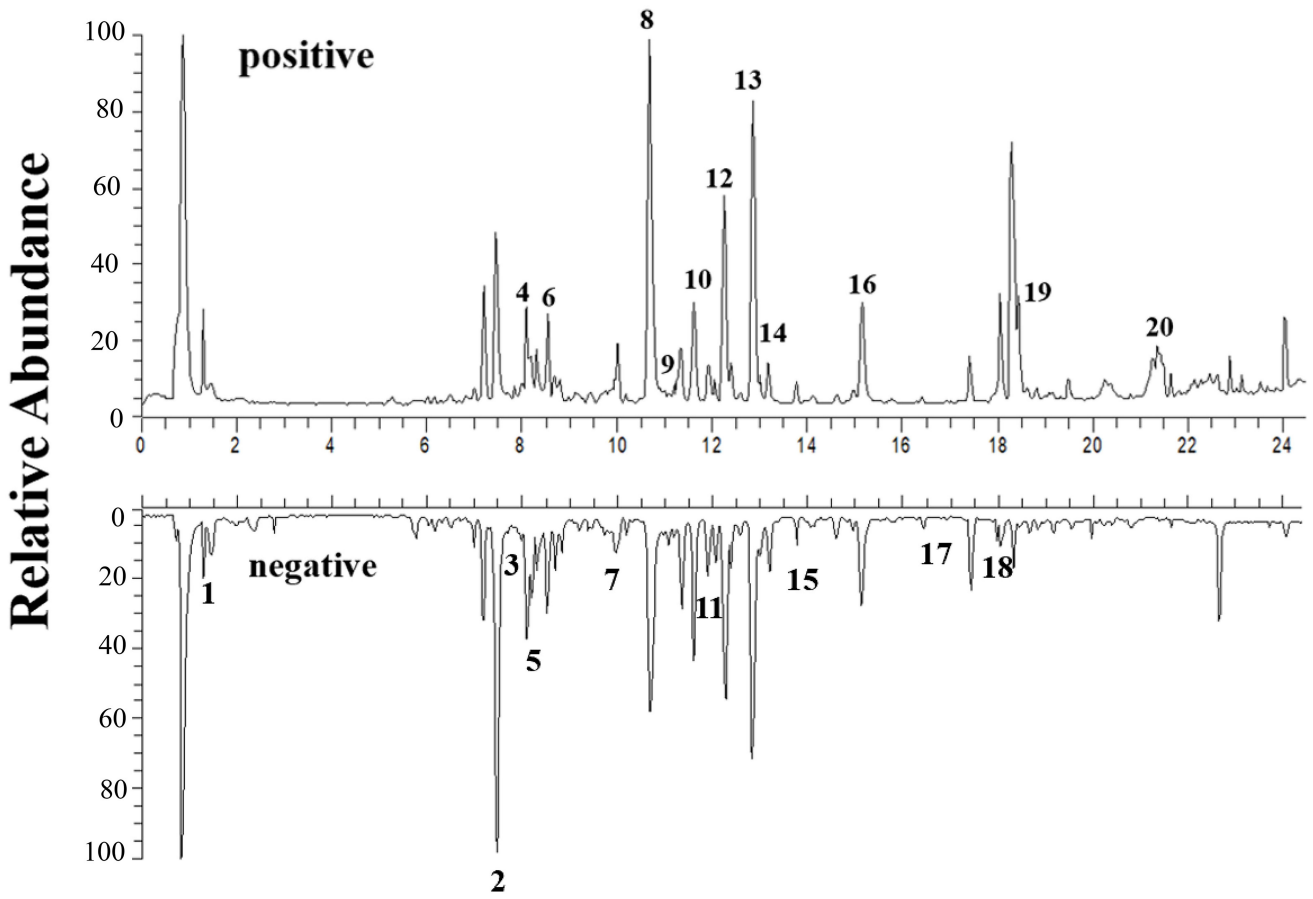
The total ion current chromatogram in positive and negative ion mode for HQD.

The chemical components in HQD were detected by UPLC-MS for quality control. Moreover, the lyophilized extract powder of HQD was prepared in the same batch for animal administration to ensure consistent quality. A total of 20 compounds were identified (Table 1), which are similar to those reported in other studies (Shibano et al., 2010; Du et al., 2020; Tang et al., 2022), including flavonoids such as baicalein, baicalin, and wogonin; glycosides such as albiflorin; and some acids such as benzoic acid. However, the main chemical component glycyrrhizic acid in *licorice* was not identified in this study. The reason may be that the proportion of *licorice* in HQD is low, and the extraction content is too low to be detected (Figure 2).

**Table 1:** Compounds identified in HQD.

**No**.	**Compounds**	**RT (min)**	**Ion**	**m/z**	**Formula**
1	Methylmalonic acid	1.476	[M-H]^−^	117.0194	C_4_H_6_O_4_
2	Albiflorin	7.459	[M-H]^−^	479.1562	C_23_H_28_O_11_
3	Suberic acid	7.916	[M-H]^−^	173.0820	C_8_H_14_O_4_
4	Scutellarin	8.309	[M+H]^+^	463.0868	C_21_H_18_O_12_
5	Quercetin	8.377	[M-H]^−^	301.0356	C_15_H_10_O_7_
6	Dibenzylamine	8.544	[M+H]^+^	198.1275	C_14_H_15_N
7	Luteolin	9.993	[M-H]^−^	285.0406	C_15_H_10_O_6_
8	Baicalein	10.684	[M+H]^+^	271.0592	C_15_H_10_O_5_
9	Kaempferol	11.194	[M+H]^+^	287.0556	C_15_H_10_O_6_
10	Baicalin	11.622	[M+H]^+^	447.0919	C_21_H_18_O_11_
11	3-tert-Butyladipic acid	11.894	[M-H]^−^	201.1134	C_10_H_18_O_4_
12	(2S,3S,4S,5R,6S)-3,4,5-trihydroxy-6- [(5-hydroxy-8-methoxy-4-oxo-2-phenyl-4H-chromen-7-yl)oxy]oxane-2-carboxylic acid	12.263	[M+H]^+^	461.1076	C_22_H_20_ O_11_
13	Wogonin	12.846	[M+H]^+^	285.0750	C_16_H_12_O_5_
14	5,7-dihydroxy-3,8-dimethoxy-2-phenyl-4H-chromen-4-one	13.192	[M+H]^+^	315.0853	C_17_H_14_O_6_
15	Benzoic acid	13.772	[M-H]^−^	121.0288	C_7_H_6_O_2_
16	5,7,8-Trihydroxyflavone	15.140	[M+H]^+^	271.0598	C_15_H_10_O_5_
17	Dodecanedioic acid	16.451	[M-H]^−^	229.1446	C_12_H_22_O_4_
18	Chrysin	18.112	[M-H]^−^	253.0508	C_15_H_10_O_4_
19	Dodecylamine	18.465	[M+H]^+^	186.2214	C_12_H_27_N
20	Dibutyl phthalate	21.337	[M+H]^+^	279.1588	C_16_H_22_O_4_

### HQD Improves the Motor Coordination Abilities of Rats With PD Induced by Rotenone

**Figure 3:**
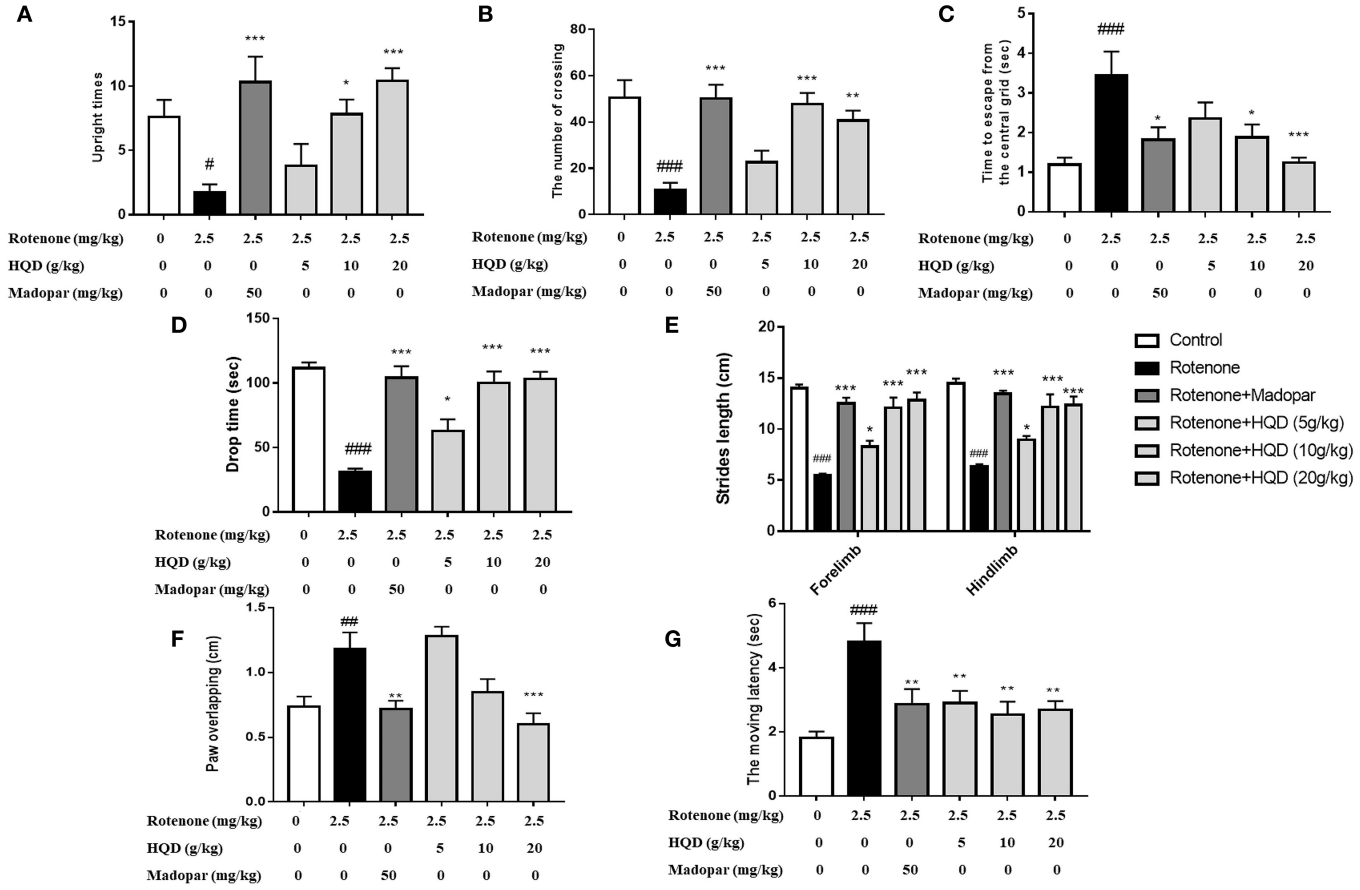
The improvement effects of HQD on motor coordination abilities of rotenone-induced PD rats. **(A)** Upright times in open field. **(B)** The number of crossing in open field. **(C)** Time to escape from the central grid in open field. **(D)** Drop time from the rotating rod. **(E)** Strides length in gait experiment. **(F)** Paw of overlapping in gait experiment. **(G)** The moving latency in grid experiment. ^#^*P* < 0.05, ^##^*P* < 0.01, ^###^*P* < 0.001 vs. control group; *P < 0.05, **P < 0.01, ***P < 0.001 vs. rotenone group (*n* = 6–10).

Open field test, rotating rod experiment, gait, and grid experiment were used to observe the improvement effect of HQD on the motor coordination abilities of rotenone-induced PD rats. The results showed that the upright times (*P* < 0.05) and the number of crossing (*P* < 0.001) in the open field were significantly reduced in the rotenone group, and the residence time (*P* < 0.001) in the central grid was significantly increased as compared with the control group (Figures 3A–C). HQD (10 and 20 g/kg) significantly increased the upright times (*P* < 0.05, *P* < 0.001) and the number of crossing (*P* < 0.001, *P* < 0.01) and significantly reduced the residence time in the central grid (*P* < 0.05, *P* < 0.001) in rotenone-induced PD rats (Figures 3A–C), suggesting that HQD obviously improved the autonomous activities of rats with PD.

In the rotenone group, the fall time of rats from the rotating rod (*P* < 0.001) was markedly shortened (Figure 3D), the stride length of rats was significantly reduced (*P* < 0.001), the degree of paw overlapping (*P* < 0.01) was significantly increased (Figures 3E,F), and the moving latency (*P* < 0.001) in the grid experiment increased as compared with the control group (Figure 3G). After administration of HQD (20 g/kg), the drop time (*P* < 0.001) of rats with PD was significantly extended (Figure 3D), the stride length (*P* < 0.001) of rats with PD was significantly increased, the degree of paw overlapping (*P* < 0.001) was significantly reduced (Figures 3E,F), and the moving latency (*P* < 0.01) was significantly reduced (Figure 3G) compared with the rotenone group. The above results indicate that HQD could improve the motor coordination abilities of rats with PD induced by rotenone.

### HQD Improves the Muscle Strength of Rats With PD Induced by Rotenone

**Figure 4:**
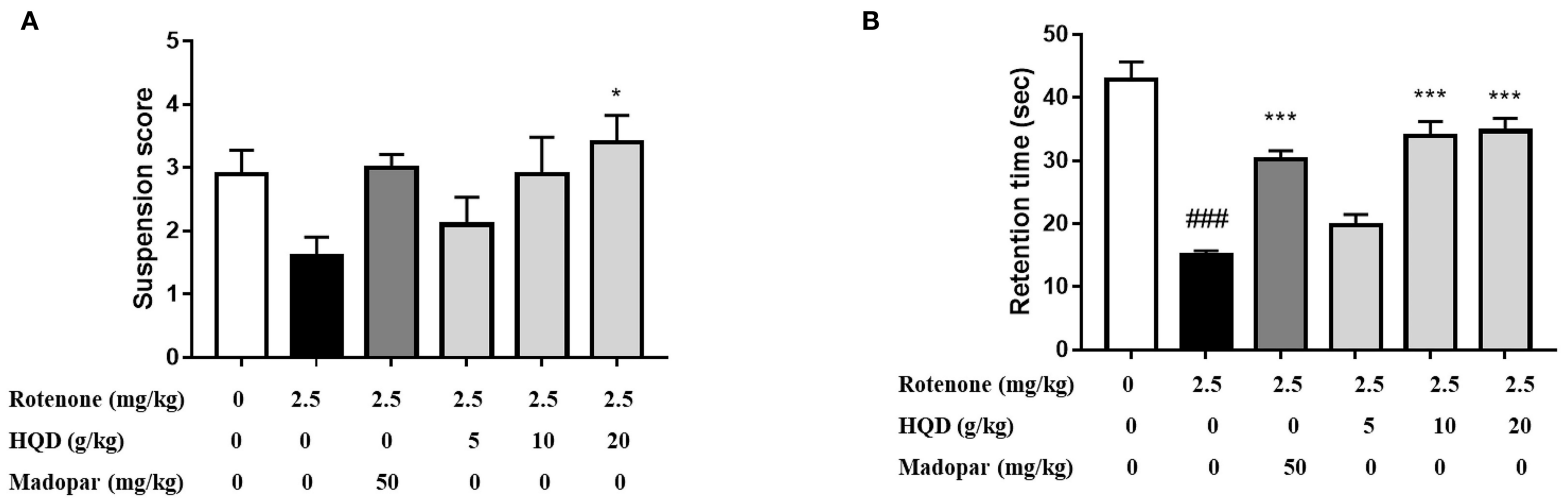
The improvement effects of HQD on muscle strength of rotenone-induced PD rats. **(A)** Suspension score in suspension test. **(B)** The retention time in inclined plate test. ^###^*P* < 0.001 vs. control group; *P < 0.05, ^#x0002A;**^P < 0.001 vs. rotenone group (*n* = 10).

Suspension and inclined plate tests were used to observe the effects of HQD on the muscle strength of rats with PD induced by rotenone. The results showed that the suspension score was reduced (Figure 4A), and the retention time (*P* < 0.001) in the inclined plate was significantly shortened in rats of the rotenone group (Figure 4B). After administration of HQD (20 g/kg), the suspension score (*P* < 0.05) was significantly increased (Figure 4A), and the retention time (*P* < 0.001) was obviously increased (Figure 4B) compared with the rotenone group. The above results indicate that HQD can improve the muscle strength of rats with PD induced by rotenone.

### HQD Increases the Number of TH-Positive Neurons and Alleviates Pathological Changes

**Figure 5:**
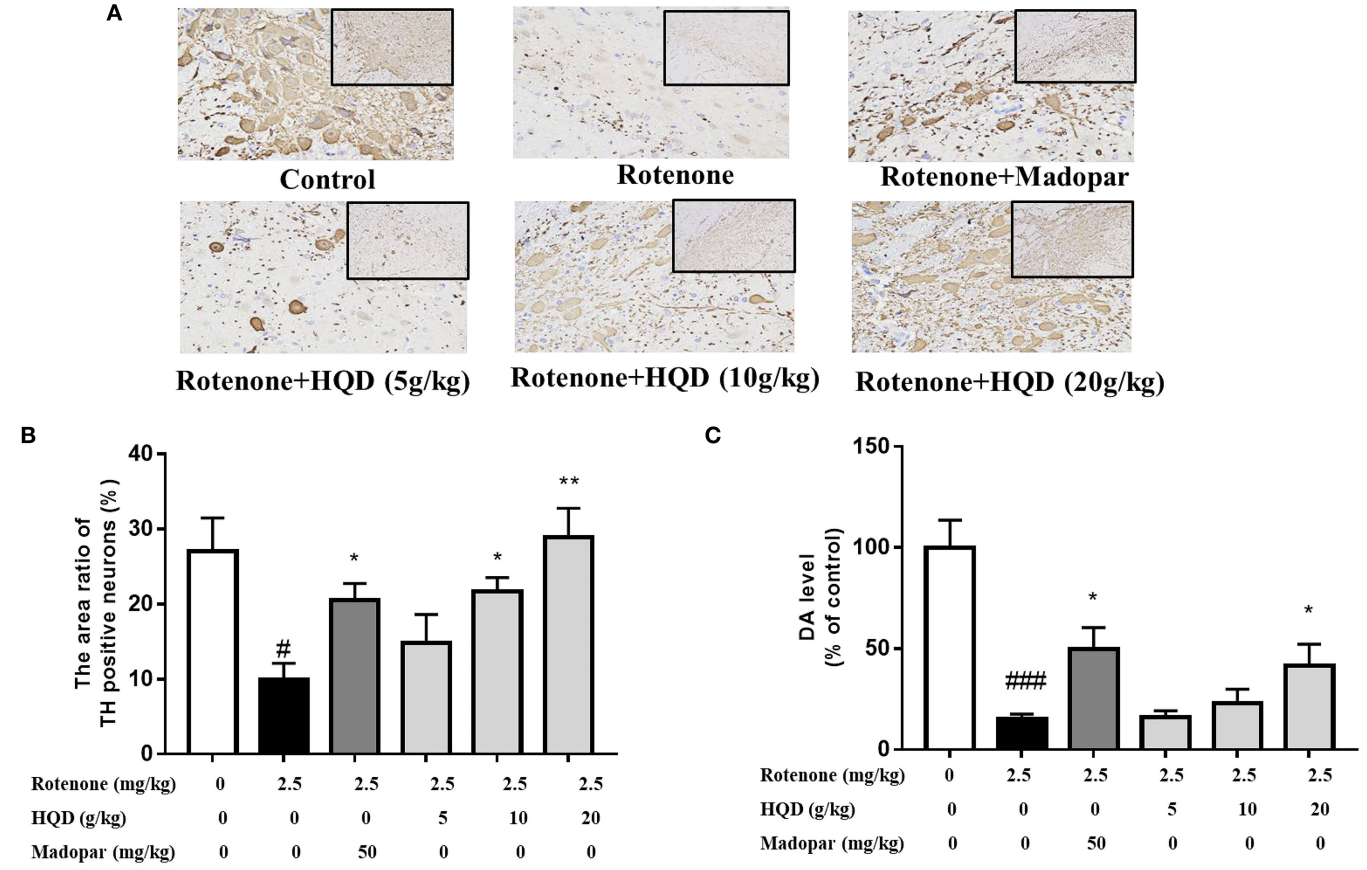
Influence of HQD on TH-positive neurons in SN and dopamine level in striatum. **(A)** Representative microphotographs of TH-immunostaining neurons in the SN. **(B)** Quantification of the area ratio of TH-positive neurons (*n* = 3). **(C)** The dopamine content in striatum (*n* = 6). ^#^*P* < 0.05, ^*###*^*P* < 0.001 vs. control group; ^*^P < 0.05, ^**^P < 0.01 vs. rotenone group.

Immunohistochemistry was performed to evaluate the effect of HQD on dopaminergic neurons in SN (Haddadi et al., 2020). The TH-immunopositive neurons and protrusions in SN of the control group were clearly visible, and the somata and fibers of DA neurons were deeply stained (Figure 5A). The number of TH-immunopositive neurons in SN of the rotenone group was significantly decreased (*P* < 0.05) compared with the control group, while HQD (10 and 20 g/kg) can significantly increase (*P* < 0.05, *P* < 0.01) the number of TH-positive neurons (Figure 5B) compared with the rotenone group.

The pathological changes of SN in PD rats were also evaluated. The number of neuronal cells in SN region of control rats was relatively abundant and evenly distributed with normal cell morphology. Compared with the control rats, the unclear boundary between cytoplasm and nucleus and irregular cell shape was observed in the rotenone group. Compared with the rotenone group, the shape of cells was more regular and the distribution was more uniform after HQD administration (Supplementary Figure).

### HQD Mitigates the Loss of Dopamine Content

The main pathological change of PD is the degeneration and death of dopaminergic neurons in SN of the midbrain, which leads to the significant decrease of DA content in the striatum. The effect of HQD on DA content in the striatum was determined. The DA content of the rotenone group was significantly (*P* < 0.001) decreased as compared with the control group. However, compared with the rotenone group, administration of 20 g/kg HQD could significantly increase (*P* < 0.05) the content of DA in the striatum of rats with PD, while 5 and 10 g/kg HQD had no significant effect on increasing DA content (Figure 5C).

### HQD Attenuates Rotenone-Induced Mitochondrial Dysfunction

**Figure 6:**
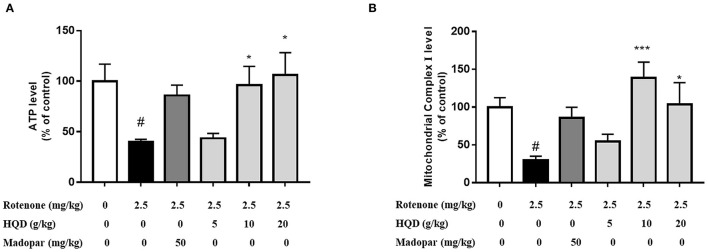
Effect of HQD on mitochondrial function of rotenone-induced PD rats. **(A)** The content of ATP in cerebral cortex. **(B)** The activity of mitochondrial complex I in cerebral cortex. ^#^*P* < 0.05 vs. control group; ^*^P < 0.05, ^***^P < 0.001 vs. rotenone group (*n* = 6).

Mitochondrial dysfunction is accompanied by the decrease of mitochondrial complex I activity. When the function of complex I is impaired, the production of reactive oxygen species increased. A large amount of reactive oxygen caused lipid peroxidation in mitochondrial membrane and damaged mitochondrial DNA, therefore, weakening or failing oxidative phosphorylation, which leads to the reduced production of ATP rapidly. The effects of HQD on mitochondrial dysfunction in rats with PD were evaluated. The results indicate that compared with the control group, the ATP level (*P* < 0.05) and the activity of mitochondrial complex I (*P* < 0.05) in the rotenone group were significantly decreased (Figures 6A,B). Compared with the rotenone group, the levels of ATP (P < 0.05, P < 0.05) and mitochondrial complex I (*P* < 0.001, *P* < 0.05) were significantly higher after treatment with HQD (10 and 20 g/kg), and 5 g/kg HQD had no such effect, indicating that HQD (10 and 20 g/kg) could improve mitochondrial dysfunction in rats with PD.

### Mitochondrial Metabolomics Study

**Figure 7:**
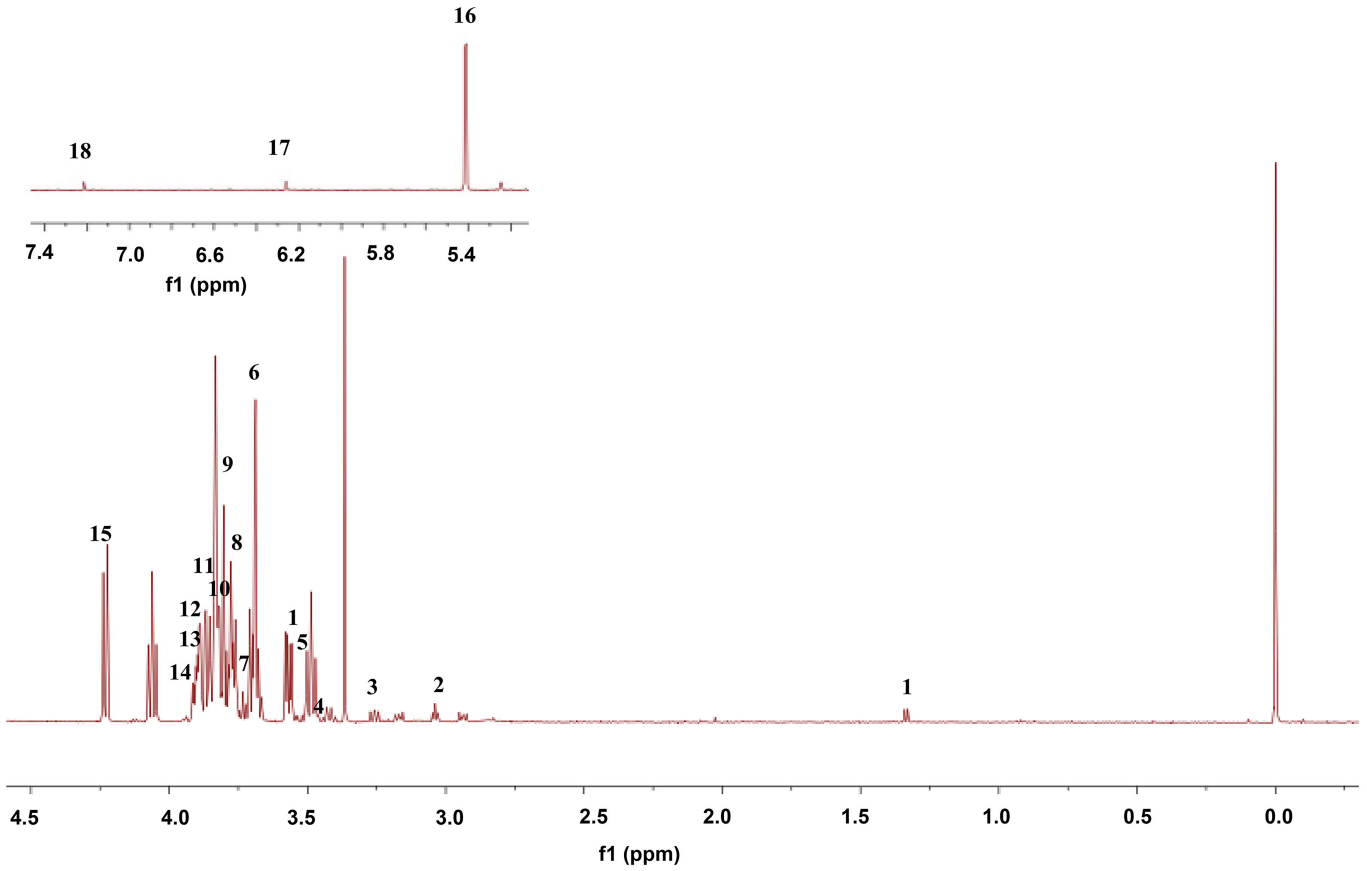
Representative ^1^H-NMR spectra of mitochondria in midbrain in control rats. The numbers 1–18 are consistent with those in Table 2.

The ^1^H-NMR spectra of mitochondria in the midbrain of control rats are shown in Figure 7. Eighteen metabolites were identified by referring to previous literatures and chemical shifts recorded in ^1^H-NMR databases, including Human Metabolomics Database and Biological Magnetic Resonance Data Bank, as shown in Table 2. Eighteen metabolites mainly include threonine, lysine acid, arginine, AcAc, and glycine. Among them, AcAc is a ketone body, and the content of AcAc in mitochondrial of the midbrain is significantly higher than the control group. It was found that ketone bodies are closely related to mitochondrial function, which can improve the pathological damage of dopaminergic neurons and mitochondrial function of neurons in mice with PD.

**Table 2:** Distribution of ^1^H-NMR peaks in control rats.

**No**.	**Metabolite**	**Chemical shift (ppm)**
1	Threonine	1.34 (d), 3.58 (d)
2	Lysine acid	3.03 (t)
3	Arginine	3.26 (t)
4	Acetoacetic acid	3.44 (s)
5	Glycine	3.54 (s)
6	Dimethylglycine	3.71 (s)
7	Acetylcholine	3.73 (t)
8	Glutamate	3.76 (m)
9	Glutamine	3.78 (m)
10	Guanidoacetic acid	3.79 (s)
11	N-Methyl-a-aminoisobutyric acid	3.85 (s)
12	3-Mercaptopyruvic acid	3.86 (d)
13	N-Acetylserine	3.88 (m)
14	Vinylacetylglycine	3.91 (d)
15	Guanosine diphosphate	4.23 (dd)
16	Linoleic acid	5.41 (d)
17	Acrylamide	6.23 (m)
18	5-Hydroxyindoleacetic acid	7.22 (s)

### HQD Regulated Metabolic Abnormality in Rotenone-Induced PD Rats

To further study the changes in metabolites after HQD treatment, partial least squares-discriminant analysis (PLS-DA) was used for analysis and model verification. The differential metabolites were identified by combining the S-plot with the VIP value.

**Figure 8:**
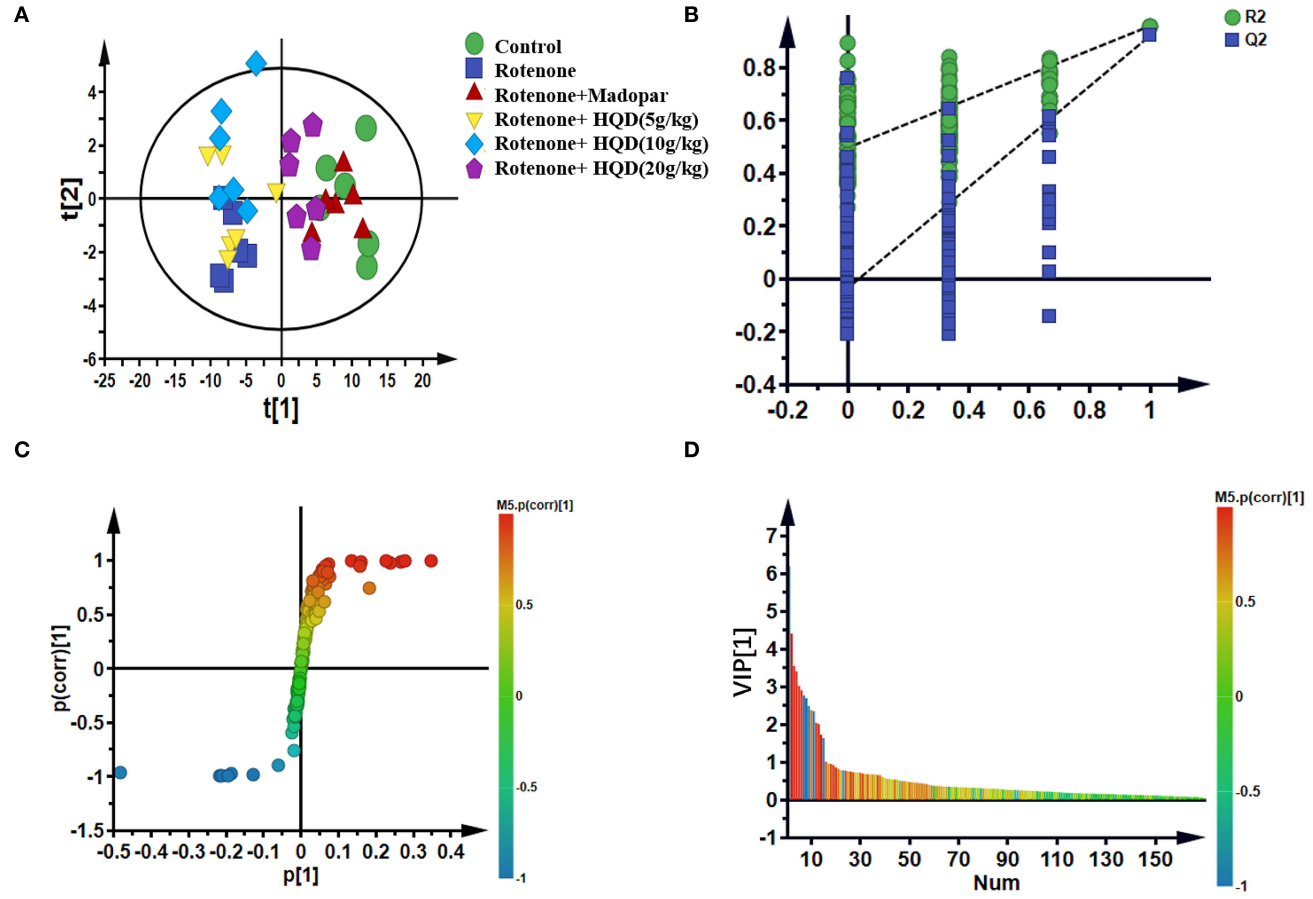
Multivariate statistical analysis of metabolites in mitochondria of midbrain among control group, rotenone group, rotenone + HQD groups, and rotenone + madopar group. **(A)** PLS-DA score plot. **(B)** Model validation plot of PLS-DA. **(C)** S-plot of OPLS-DA. **(D)** VIP values of OPLS-DA.

**Figure 9:**
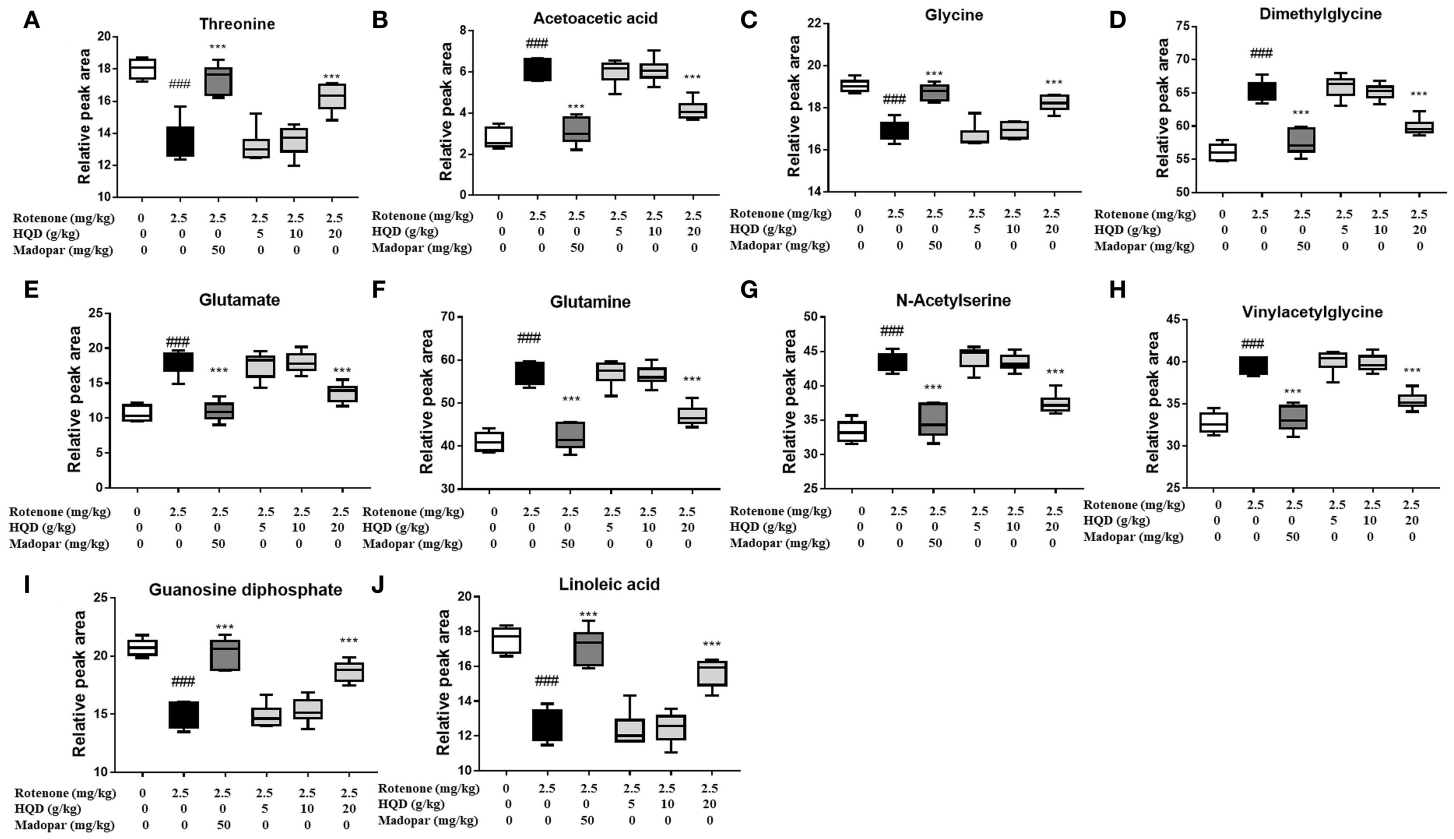
Relative peak areas of the differential metabolites affected by HQD in rats with PD: **(A)** threonine; **(B)** acetoacetic acid; **(C)** glycine; **(D)** dimethylglycine; **(E)** glutamate; **(F)** glutamine; **(G)** N-acetylserine; **(H)** vinylacetylglycine; **(I)** guanosine diphosphate; **(J)** linoleic acid. ^###^*P* < 0.001 vs. control group; ^***^P < 0.001 vs. rotenone group (*n* = 6).

In PLS-DA scatterplot (Figure 8A), the control group and the rotenone group were obviously separated, and HQD (20 g/kg) and madopar group were close to the control group, suggesting that HQD has regulatory effects on metabolic profile of rats with PD. The values of parameters *R*^2^ and *Q*^2^ of the PLS-DA model [*R*^2^ (X) = 0.862, *Q*^2^ (Y) = 0.922] were smaller than the primary values, which indicates that the model has preeminent prediction ability (Figure 8B). The differential metabolites were determined by S-plot and VIP values, and the relative peak areas were performed by independent *t* testing (Figures 8C,D). Compared to the control group, rotenone treatment can downregulate 4 differential metabolites (glycine, threonine, guanosine diphosphate, and linoleic acid) and upregulate 6 differential metabolites (AcAc, dimethylglycine, glutamate, glutamine, N-acetylserine, and vinylacetylglycine) (*P* < 0.001). After treatment of HQD (20 g/kg), the contents of these 10 differential metabolites can be significantly regulated (*P* < 0.001; Table 3; Figure 9).

**Table 3:** Differential metabolites regulated by HQD in rotenone-induced PD rats.

**No**.	**Metabolite**	**Rotenone vs**.	**HQD (20 g/kg)**
		**control**	**vs. rotenone**
1	Threonine	**↓^###^**	↑^***^
2	Acetoacetic acid	**↑^###^**	↓^***^
3	Glycine	**↓^###^**	↑^***^
4	Dimethylglycine	**↑^###^**	↓^***^
5	Glutamate	**↑^###^**	↓^***^
6	Glutamine	**↑^###^**	↓^***^
7	N-Acetylserine	**↑^###^**	↓^***^
8	Vinylacetylglycine	**↑^###^**	↓^***^
9	Guanosine diphosphate	**↓^###^**	↑^***^
10	Linoleic acid	**↓^###^**	↑^***^

### *P < 0.001 rotenone vs. control group*.

*** *P < 0.001 HQD (20 g/kg) vs. rotenone group*.

### HQD Regulates Aerobic Glycolysis Pathway Instead of Ketone Body Pathway to Relieve PD Symptoms

From our metabolomics results, we found that the content of AcAc, a kind of ketone body, was significantly increased as compared with the control group. Since ketone bodies were closely related to the neurodegenerative diseases (Ding et al., 2013a,b; Shi et al., 2015; Vidali et al., 2015; Hasan-Olive et al., 2019; Petrick et al., 2020), we chose AcAc as the breakthrough point for follow-up research.

**Figure 10:**
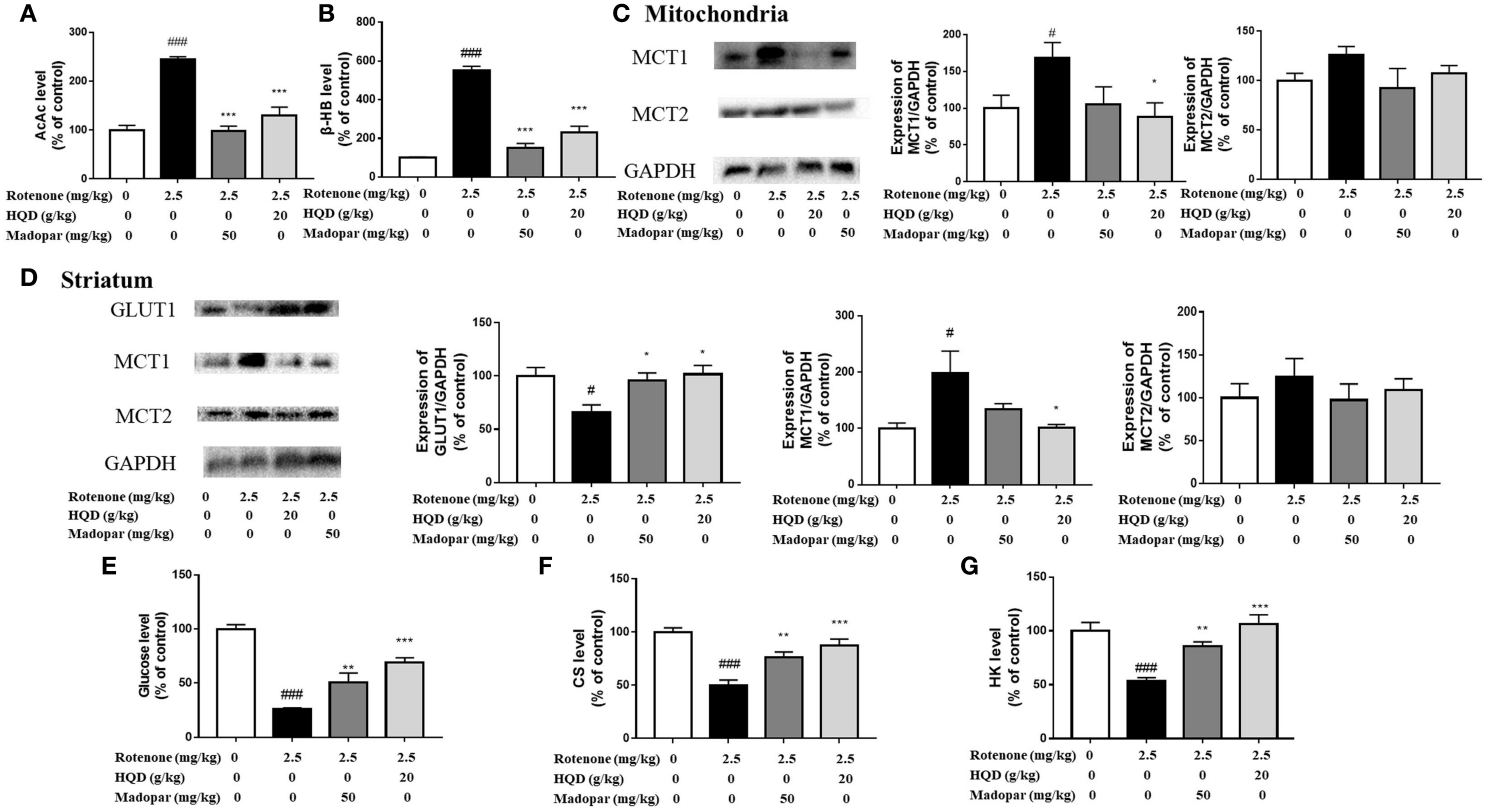
Effect of HQD on aerobic glycolysis pathway and ketone body pathway. **(A)** The effect of HQD on AcAc level in midbrain mitochondria of rats with PD (*n* = 6). **(B)** The effect of HQD on β-HB level in midbrain mitochondria of rats with PD (*n* = 6). **(C)** The expression levels of MCT1 and MCT2 in midbrain mitochondria of rats with PD (*n* = 4). **(D)** The expression levels of GLUT1, MCT1, and MCT2 in striatum of rats with PD (*n* = 4). **(E)** The effect of HQD on glucose level in striatum of rats with PD (*n* = 6). **(F)** The effect of HQD on CS level in midbrain mitochondria of rats with PD (*n* = 6). **(G)** The effect of HQD on HK level in striatum of rats with PD (*n* = 6). ^#^*P* < 0.05, ^*###*^*P* < 0.001 vs. control group; ^*^P < 0.05, ^**^P < 0.01, ^***^P < 0.001 vs. rotenone group.

To explore the potential mechanism of HQD in regulating ketone bodies, the content of key metabolite AcAc in midbrain mitochondria was determined, and the content of the ketone body β-HB was further detected. The results showed that the contents of ketone bodies (AcAc and β-HB) were significantly increased in the rotenone group (*P* < 0.001, *P* < 0.001) compared with the control group, while HQD treatment markedly decreased the contents of AcAc and β-HB (*P* < 0.001, *P* < 0.001) (Figures 10A,B) compared with the rotenone group. MCT1 and MCT2 were responsible for transporting ketone bodies from outside to the brain through the blood–brain barrier. The results showed that the expression of MCT1 in midbrain mitochondria (*P* < 0.05) and striatum (*P* < 0.05) obviously increased in the rotenone group compared with the control group, but decreased after HQD treatment (*P* < 0.05, *P* < 0.05) (Figures 10C,D) compared with the rotenone group. However, there was no significant change in the expression of MCT2 in midbrain mitochondria and striatum (Figures 10C,D).

The above results suggested that the content of ketone body compensatively increased in the rotenone group, indicating that the ketone body pathway was activated. Studies have shown that when glucose supply is insufficient, ketone bodies are used as an alternative energy source (Ding et al., 2013a,b). We speculate that the activation of the ketone body pathway may be attributed to the blockade of the aerobic glycolysis pathway. The aerobic glycolysis pathway occurs in the cytoplasmic matrix. Glucose crosses the blood–brain barrier with the assistance of glucose transporter GLUT1 and is transported to neurons, further converted into glucose 6-phosphate under the action of HK, and then converted into pyruvate through a series of processes and further transformed into acetyl-CoA in mitochondria. Under the action of CS, ATP is finally synthesized through the TCA cycle for brain use. Since glucose could not enter mitochondria, the content of glucose transporter GLUT1 and the content of glucose in the striatum were detected, which found that the expression of GLUT1 (*P* < 0.05) and the content of glucose (*P* < 0.001) decreased significantly in the rotenone group compared with the control group, but increased significantly after HQD treatment (*P* < 0.05, *P* < 0.001) (Figures 10D,E) compared with the rotenone group. Furthermore, we examined the content of the mitochondrial tricarboxylic acid cycle rate-limiting enzyme CS. It was found that the content of CS decreased in mitochondria of the rotenone group (*P* < 0.001) compared with the control group, but after HQD treatment, the content of CS increased obviously (*P* < 0.001; Figure 10F) compared with the rotenone group. Furthermore, HK, the first key enzyme in the process of glucose decomposition, was determined in the striatum, and it was found that HK decreased significantly in the rotenone group (*P* < 0.001) compared with the control group, but increased significantly after administration of HQD (*P* < 0.001; Figure 10G) compared with the rotenone group. The results indicated that the decrease in glucose content was parallel to the increase of substitute substrate (ketone body) in the rotenone group. HQD could improve the mitochondrial function of rats with PD by upregulating the expression of glucose transporter GLUT1, the level of HK, and the content of tricarboxylic acid cycle rate-limiting enzyme CS to reactivate the aerobic glycolysis pathway.

## Discussion

Parkinson's disease is a common neurodegenerative disease, and its incidence is about 1–2% in people over 60 years (Song et al., 2014). PD is accompanied by behavioral changes, such as resting tremor and muscle rigidity, and is life-threatening in old patients. In this experiment, a variety of behavioral detection methods were used to comprehensively evaluate the behavioral changes of rotenone-induced PD rats. The decrease in coordination ability is an important behavioral characteristic of patients with PD, and the improvement of coordination is an important criterion for assessing the therapeutic effects of anti-PD drugs. The experiments of open field, rotating rod, gait, and grid showed that HQD can obviously improve the motor coordination ability of rats with PD. The decrease in muscle strength is also an important behavioral change in patients with PD. Suspension and inclined plate experiments proved that HQD can improve the muscle strength of rats with PD.

Mitochondria are dynamic organelles, which play an important role in various physiological processes in cells to maintain the integrity of neural circuits. Mitochondrial homeostasis imbalance can lead to the development of progressive pathological conditions related to aging and neurodegeneration. The biological characteristics of PD are protein misfolding and massive neuronal death. The pathological mechanism of these biological characteristics is highly complex, including metabolic and signal transduction dysfunction. Dopaminergic neurons in SN are metabolically active cells with large volume, extensive dendritic structure, and calcification activity, so they are particularly vulnerable to energy deficiency. Mitochondria are the main place for generating energy (Dias et al., 2013; Poewe et al., 2017). Therefore, it is of great significance to find a new target for treating PD from the perspective of mitochondria.

Rotenone is an inhibitor of mitochondrial complex I (Heikkila et al., 1985). Studies have shown that mitochondrial dysfunction leads to the chronic production of reactive oxygen species (ROS) and the death of dopaminergic neurons (Thakur and Nehru, 2013; Chao et al., 2018). The decline of mitochondrial function, especially the decrease of ATP production caused by the change of mitochondrial complex I activity, is involved in the pathological process of PD (Perier and Vila, 2012). Interestingly, HQD treatment can significantly increase the level of ATP, and restore the activity of mitochondrial complex I. The results indicated that HQD could improve mitochondrial dysfunction in rotenone-induced PD rats.

Studies have shown that metabolites involved in the TCA cycle, energy metabolism, glycolysis, and redox metabolism, show different levels in whole cells and mitochondria (Chen et al., 2016). Whole-cell metabolite spectrum analysis cannot reflect the real dynamic changes of mitochondrial metabolism. Mitochondrial metabolomics can directly find out the potential functional changes of various metabolic pathways and signal networks at a subcellular level, which is necessary for elucidating the structure and quantitative analysis of mitochondrial metabolites. In addition, mitochondrial diseases are more common than expected, which are caused by genetic defects of mitochondrial metabolic enzymes, and then lead to ATP synthesis disorder and energy shortage (Schaefer et al., 2004; Bonnen et al., 2013; Nesti et al., 2015; Tosserams et al., 2018). Due to the high incidence of mitochondrial dysfunction in many diseases, especially neurodegenerative diseases (Lebedeva et al., 2003; Hsu et al., 2013; Gao et al., 2015b; Yi et al., 2015; Barrera et al., 2016; Kudryavtseva et al., 2016; Cormio et al., 2017; Ristic et al., 2017), it is urgent to study and clarify mitochondrial metabolic changes caused by diseases. In this study, ^1^H-NMR metabolomics combined with multivariate statistical analysis was used to analyze the changes of endogenous metabolites in midbrain mitochondria of rats with PD (Johnson et al., 2016; Kumari et al., 2020). It was found that the content of AcAc, a kind of ketone body as an alternative energy source for glucose, was significantly increased after rotenone injection, which attracted our attention for subsequent research.

Glucose is the main energy substrate of brain metabolism (Leino et al., 2001), which can pass through the blood–brain barrier through GLUT1 and be transported to neurons to be converted into pyruvate through glycolysis reaction, and finally converted into ATP through tricarboxylic acid cycle in mitochondria for brain utilization (Gano et al., 2014). When glucose energy supply is insufficient, the brain can also use ketone bodies, such as β-HB and AcAc, as main fuels, which cross the blood–brain barrier through MCT1 and MCT2 and are further converted into ATP for utilization by the brain (Zhang and Xie, 2017; Zhang et al., 2018a). At this time, as an alternative energy source of glucose, the concentration of the ketone body will increase significantly (Coleman et al., 2021; Sridharan and Lee, 2021).

Ketone bodies are closely related to mitochondrial bioenergetics and mitochondrial kinetics (Veech, 2004; Thai et al., 2021). The synthesis of ketone bodies mainly occurs in the liver. In addition, astrocytes in the brain can also produce a small number of ketone bodies (Guzmán and Blázquez, 2001). Generally speaking, the uptake of ketone bodies through the blood–brain barrier is carrier-dependent, which is different from glucose transport. It does not increase with neuronal activity but is related to circulating concentration (Cunnane et al., 2016). MCT is a ketone body transporter and runs through the whole brain (Pierre and Pellerin, 2005). Studies have shown that MCT1 and MCT2 can transport ketone bodies to cross the blood–brain barrier and finally convert them into ATP for brain use.

Glucose molecules cannot pass through biofilm freely, and there is no glucose in mitochondria. However, studies have shown that GLUT1 can transport glucose to neurons and further transform it into ATP through the glycolysis pathway for brain use (Vidali et al., 2015). Studies have shown that the first step of HK-catalyzed glucose metabolism is to phosphorylate glucose into glucose 6-phosphate, which plays a key role in the aerobic glycolysis stage (Tan and Miyamoto, 2015). We found that the expression of GLUT1 decreased in the rotenone group, while the expression of GLUT1 increased in the HQD group. It has been reported in the literature that the utilization rate of β-hydroxybutyrate dehydrogenase in the brain of mice after exposure to mitochondrial toxicants increases rapidly (Tieu et al., 2003), which further leads to the increase of ketone bodies in the brain to support compensation (Yao et al., 2010; Ding et al., 2013a,b). We detected the content of ketone bodies (β-HB and AcAc) in mitochondria and the protein expression of ketone body transporters (MCT1 and MCT2). According to the experimental results, the expressions of MCT1 and MCT2 in the rotenone group increased, which indicated that the stimulation of rotenone caused the aerobic glycolysis pathway to be blocked and, therefore, transfer to the ketogenic pathway (Kuter et al., 2021). Furthermore, we found that the contents of tricarboxylic acid cycle rate-limiting enzyme (CS) and HK were decreased significantly in mitochondria of the rotenone group, but increased after HQD treatment. The results indicated that HQD improved PD symptoms through the aerobic glycolysis pathway instead of the ketone body pathway, and the activation of the aerobic glycolysis pathway was realized by upregulating the expression of glucose transporter GLUT1 and the content of tricarboxylic acid cycle rate-limiting enzyme CS and the level of HK. However, the relevant mechanisms should be verified by knockout or overexpression of key targets in the future.

**Figure 11:**
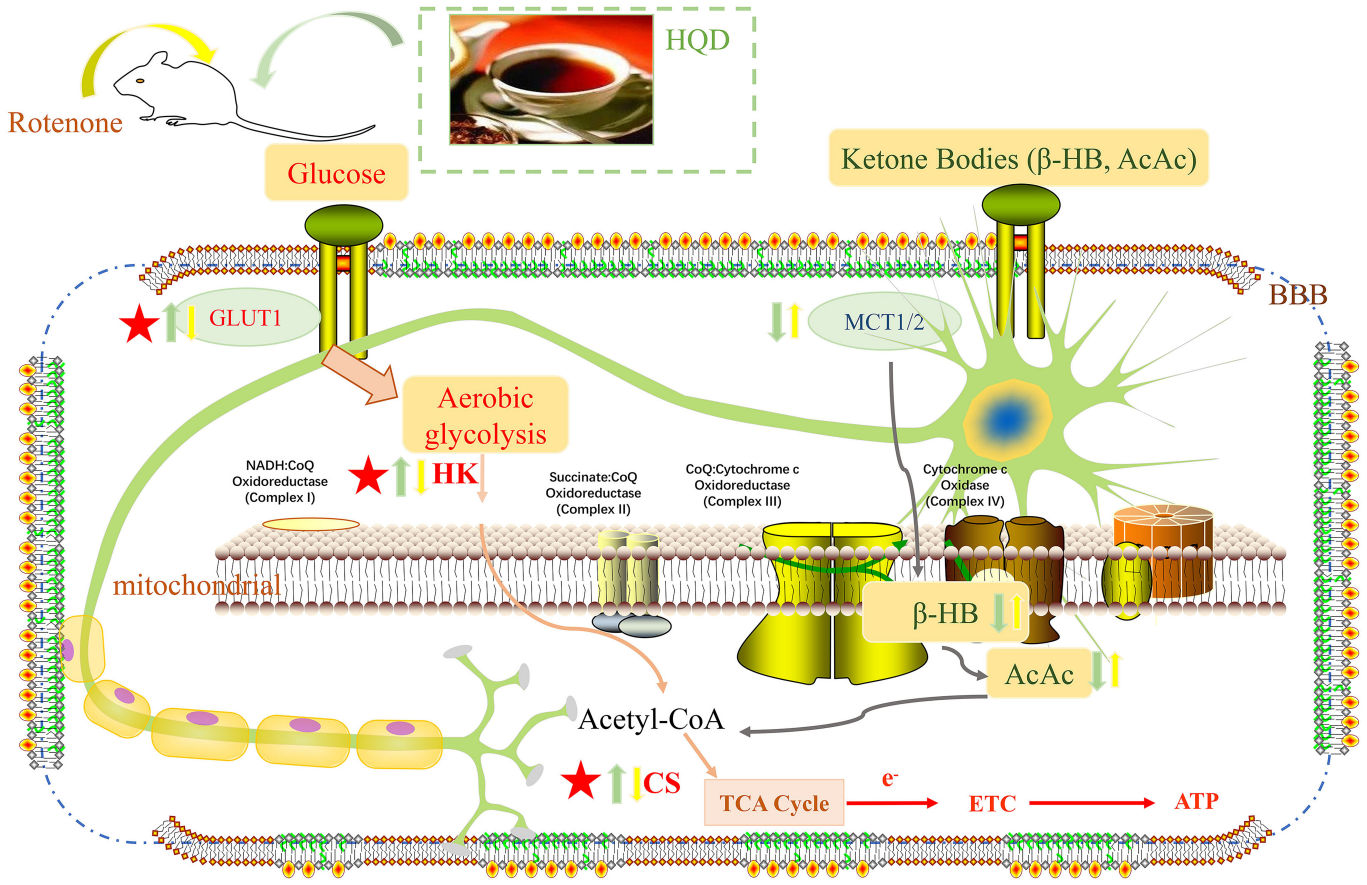
Potential mechanism of HQD for the protective effects in rotenone-induced PD rats. Yellow arrow represents the action of rotenone, green arrow represents the action of HQD, upward arrow represents up-regulation, and downward arrow represents down-regulation.

In brief, we found for the first time that HQD mainly improved rotenone-induced PD rats in terms of motor coordination and muscle strength and increased the number of TH-positive neurons and DA content. In addition, HQD can improve mitochondrial dysfunction by increasing the content of mitochondrial complex I and ATP. Besides, HQD can upregulate the expression of glucose transporter GLUT1 and the content of tricarboxylic acid cycle rate-limiting enzyme CS and the level of HK to activate the aerobic glycolysis pathway (Figure 11).

## Data Availability Statement

The original contributions presented in the study are included in the article/Supplementary Material, further inquiries can be directed to the corresponding authors.

## Ethics Statement

The animal study was reviewed and approved by the Animal Ethics Committee of Shanxi University.

## Author Contributions

LG and MC provided the concept, designed the study, and participated in data analysis. MC performed the experiments and drafting of the manuscript. X-mQ, G-hD, and LG provided oversight. LG contributed to revising and proofreading the manuscript. All authors read and approved the final manuscript and agree to be accountable for all aspects of work ensuring integrity and accuracy.

## Funding

This project was supported by the Shanxi Key Research and Development Plan Project (Grant No. 201903D321216).

## Conflict of Interest

The authors declare that the research was conducted in the absence of any commercial or financial relationships that could be construed as a potential conflict of interest.

## Publisher's Note

All claims expressed in this article are solely those of the authors and do not necessarily represent those of their affiliated organizations, or those of the publisher, the editors and the reviewers. Any product that may be evaluated in this article, or claim that may be made by its manufacturer, is not guaranteed or endorsed by the publisher.

## Supplementary Material

Supplementary Figure The effect of HQD on pathological changes of brain tissue in rats with PD.

## Abbreviations

PD: Parkinson's disease
HQD: Huangqin Decoction
SN: substantia nigra
MPTP: N-methyl-4-phenyl-1,2,3,6-tetrahydropyridine
DA: dopamine
β-HB: β-hydroxybutyric acid
AcAc: acetoacetic acid
CS: citrate synthase
ATP: Adenosine-triphosphate
HK: hexokinase
TSP: 3-trimethylsilyl 2,2,3,3-d4 propionate
UPLC-MS: ultra performance liquid chromatography coupled with mass spectrometry
ESI: electrospray ionization
TH: Tyrosine Hydroxylase.
