# Supplementary figures and images for: Huangqin Decoction Exerts Beneficial Effects on Rotenone-Induced Rat Model of Parkinson's Disease by Improving Mitochondrial Dysfunction and Alleviating Metabolic Abnormality of Mitochondria
Source: Front Aging Neurosci. 2022 Jul 15;14:911924. doi: 10.3389/fnagi.2022.911924 (PMC9334858; doi:10.3389/fnagi.2022.911924)

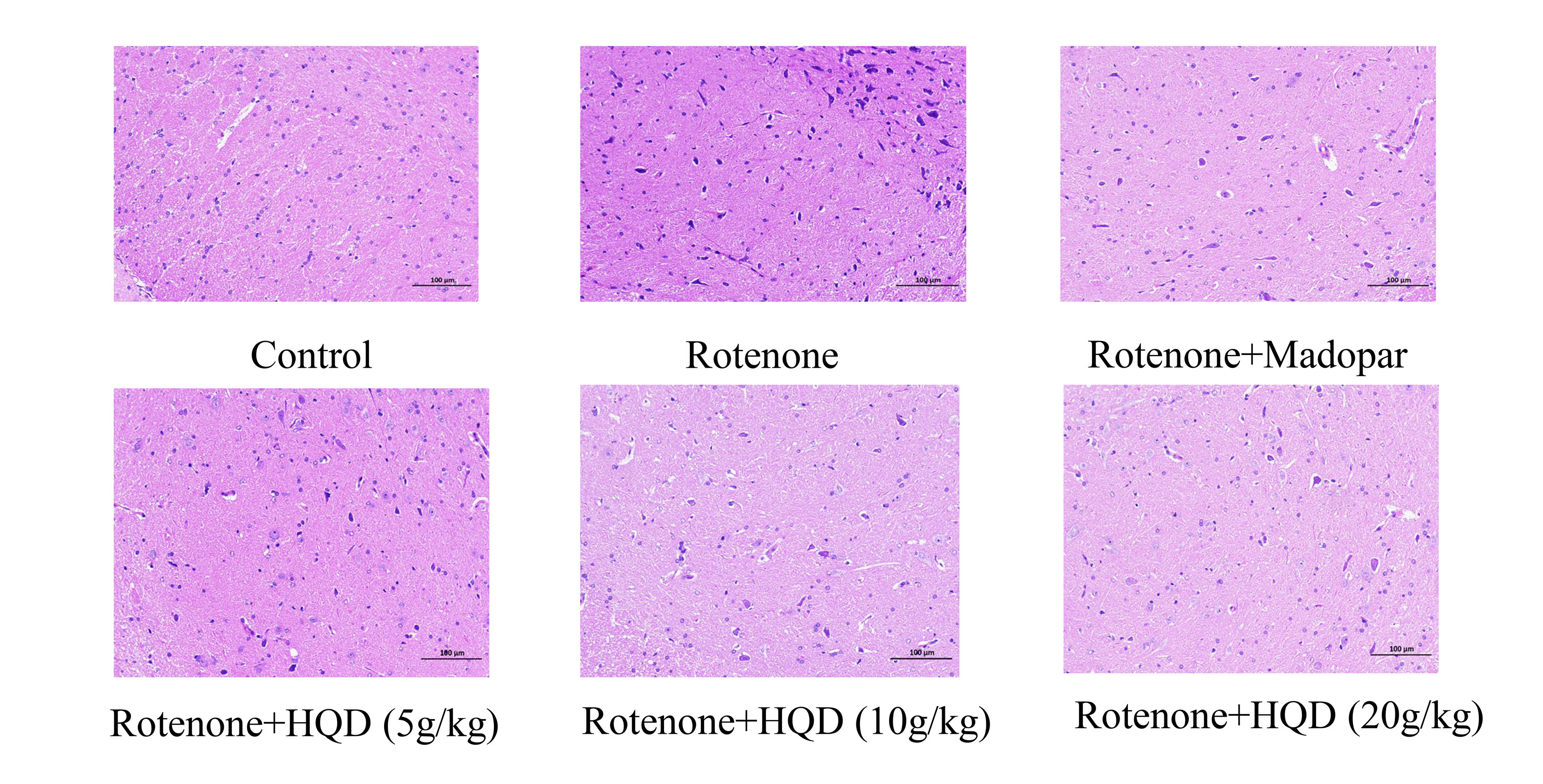

Supplement: Supplementary Figure — The effect of HQD on pathological changes of brain tissue in rats with PD. [file Image_1.JPEG]
